# Arboreal Mammals Are at Risk Under Climate Change: Insights From a Global Systematic Review and Meta‐Analysis

**DOI:** 10.1111/gcb.71034

**Published:** 2026-08-08

**Authors:** Dinayadura Kalathma Navanjalie Samanthi De Silva, Carly Campbell, Grant Hamilton

**Affiliations:** ^1^ Faculty of Science, School of Biology and Environmental Science Queensland University of Technology (QUT) Brisbane Queensland Australia; ^2^ Department of the Environment, Tourism, Science and Innovation Queensland Government Brisbane Queensland Australia

**Keywords:** arboreal, biodiversity conservation, climate change, mammals, systematic literature review

## Abstract

Climate change poses significant threats to biodiversity, altering species survival and interactions within diverse ecological networks. In this systematic review, we synthesized current knowledge on climate change impacts on arboreal mammals and identified gaps, biases, and future opportunities in research. Following PRISMA guidelines, we identified 142 studies spanning 33 countries. We identified significant phylogenetic and geographic gaps in knowledge, revealing that large proportions of taxa remain unstudied or under‐represented. Australia and South America were the most frequently studied countries; however, considering the high diversity of arboreal mammals in these regions, this research focus remains inadequate. The koala and greater glider were major focal animals of climate change studies. The order *Diprotodontia* was the most studied (46%), followed by *Primates* (23%). Conversely, studies focusing on the orders *Chiroptera*, *Pilosa*, and *Microbiotheria*, as well as research conducted in Africa and Europe, were noticeably underrepresented. Extreme temperature was found to be the main driver behind many negative impacts on arboreal mammals. Meta‐analysis revealed that several arboreal mammal families are projected to experience significant future range contractions, particularly *Pseudocheiridae, Cercopithecidae*, and *Tarsiidae*. Conservation practitioners should target areas that ensure both climatic and ecological suitability for species survival in future climate scenarios, prioritising highly vulnerable species. Current research highlights the critical need for habitat protection, restoration, and management, identification of climate and thermal refugia, and conducting more species‐specific research. Future research should focus on expanding to underrepresented taxa and regions, conducting more longitudinal studies in well‐studied taxa and regions, investigating multiple climate drivers, and considering multiple species where appropriate, to enhance our understanding of climate change impacts on arboreal mammals.

## Graphical abstract

Climate change is increasingly impacting arboreal mammals, yet these effects vary across regions and taxa, and global syntheses to inform conservation priorities remain limited. This study presents the first global synthesis of climate change impacts on arboreal mammals using a systematic review, meta‐analysis, and bibliometric analysis. It identifies species‐specific risks and ecological vulnerabilities, considers the compounding effects of environmental pressures, and highlights conservation strategies. Across 142 studies conducted across 33 countries, the responses of 281 species were evaluated. The impacts were predominantly negative, with 94% of reported impacts being negative and over 90% of species being adversely affected. Australian *Diprotodontia* (46%) appeared particularly affected by climate change. *Chiroptera, Pilosa, Microbiotheria* (< 10%), and studies from Africa (6.3%) and Europe (4.9%) were underrepresented. Meta‐analysis showed an overall effect size of −49% (*p* < 0.0001), highlighting significant range contractions in arboreal mammals under future climate change. Substantial taxonomic and geographic biases persist, underscoring the future need for species‐specific research, climate refugia identification, and habitat restoration.

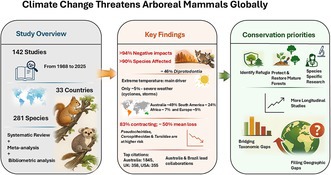

## 1 Introduction

Changes in climate already pose significant threats to global biodiversity with potentially dire consequences for species persistence, distribution, and ecosystem dynamics (IPBES 2019). Climate change and biodiversity loss reinforce one another in a feedback loop (Kim et al. 2024): as climate change accelerates species declines, the resulting loss of biodiversity weakens ecosystem resilience, making ecosystems even more susceptible to further climatic impacts (Raven and Wackernagel 2020; Weiskopf et al. 2020). When confronted with climate change, vulnerable species have three possible courses of action: to shift their distribution range, adjust to new climatic conditions, or become extinct (Hetem et al. 2014; Radchuk et al. 2019). Most species are unable to cope with current rates of climate change, as its rapid pace exceeds their ability to adapt or shift (Radchuk et al. 2019; Rubenstein et al. 2023). While climate change acts over broad spatial and temporal scales, demonstrating direct impacts of climate change can be challenging due to the complex interplay of abiotic and biotic factors, varying species vulnerabilities, and both direct (e.g., droughts, heat stress) and indirect effects (e.g., wildfires, habitat fragmentation) (Winter et al. 2016). Earth's history shows abrupt climate change as a direct or indirect driver of both minor and mass extinctions (Crowley and North 1988; Song et al. 2021). However, the risk of extinction is not just in the past. While slower, natural climate cycles have driven extinctions over millennia, today's biodiversity crisis is unfolding under the unprecedented speed and intensity of human‐induced climate change, primarily driven by greenhouse gas (GHG) emissions (e.g., Carbon dioxide (CO_2_), methane (CH_4_)) associated with fossil fuel burning, agricultural expansion, land‐use transformation, and industrial processes (Peng et al. 2026). Human‐driven climate change has raised global surface temperatures by more than 1.5°C above pre‐industrial conditions (World Meteorological Organization 2025), resulting in more frequent and intensified extreme weather events worldwide (Anderegg et al. 2020).

Local extinctions driven by climate change have already been documented across hundreds of species, with significantly higher rates observed in tropical regions compared to temperate zones (Wiens 2016). In the context of mammals, the extinction of the marine Bramble cay melomys (Waller et al. 2017), an isolated habitat specialist, marks the first recorded case of a mammal whose extinction is due to anthropogenic climate change (Fulton 2017). This event was mainly due to habitat loss resulting from climate change‐induced sea level rise (Fulton 2017; Waller et al. 2017). Studies predict that if current trends continue, up to one in six species globally may face extinction due to climate change, with regions such as Australia and South America expected to experience disproportionately high risks (Urban 2024; Urban 2015). These findings demonstrate the value of including climate change as a central consideration in biodiversity conservation research. By the end of the century, climate change is anticipated to emerge as a leading driver of biodiversity loss (CBD 2018), with potentially devastating consequences for many vulnerable species. It is a particular risk for those with specialised niches, such as arboreal mammals, whose survival is intrinsically tied to the presence of intact, connected forest canopies. Therefore, recognising climate impacts is essential to develop conservation strategies that mitigate climate change impacts on these species.

Arboreal mammals comprise a range of species, including primates (e.g., monkeys, lemurs), marsupials (e.g., koala, possums), certain carnivores (e.g., martens), and rodents (e.g., tree rats, squirrels). To thrive within their habitat, they have developed adaptations such as prehensile tails, gliding membranes, and strong, elongated limbs that support agile movement across various canopy layers (Dublin 1903). These animals are integral components of forest ecosystems, serving essential ecological functions including pollination, seed dispersal, and the maintenance of trophic balance (Lacher et al. 2019; Vieira and Izar 1999). Arboreal mammal species are particularly vulnerable to climate change, mainly due to their reliance on forested ecosystems, which are increasingly threatened by warming temperatures, wildfires, and prolonged droughts. These climatic disturbances can lead to habitat fragmentation, increased canopy openness, both of which negatively impact their mobility, and resource availability (e.g., food and habitats). Arboreal mammals also generally exhibit low litter sizes because it is more challenging for them to carry newborn offspring (Grazer and Martin 2012; Sikes and Ylönen 1998), which in turn slows their reproductive rates and limits their ability to rapidly adapt to environmental changes. Given these reasons, arboreal mammals are a group that is particularly susceptible to climate change.

A range of review articles have examined the effects of climate change across a wide range of mammals, including terrestrial (Fuller et al. 2020; Paniw et al. 2021), marine (Burek et al. 2008; Gulland et al. 2022; Learmonth et al. 2006; Moore and Huntington 2008; Orgeret et al. 2022; Ragen et al. 2008), hibernating (Findlay‐Robinson et al. 2023; Wells et al. 2022), dryland (Fuller et al. 2021), invasive (Kappes et al. 2021), large (Hetem et al. 2014), small (Griffiths and Brook 2014; Meserve et al. 2011), and mammals generally (Boutin and Lane 2014). These reviews explore a range of topics, such as the demographic responses (Paniw et al. 2021), life‐history traits (Findlay‐Robinson et al. 2023; Wells et al. 2022), health (Burek et al. 2008), fire impacts (Griffiths and Brook 2014), and potential effects (Learmonth et al. 2006). Nevertheless, there remains a notable gap in the literature specifically concerning arboreal mammals. Due to their unique ecological needs and reliance on tree‐based habitats, arboreal mammals may face distinct challenges that are not adequately explored in existing reviews. Recognising these impacts and synthesizing this information is crucial for developing conservation strategies that mitigate the effects of climate change, and for identifying gaps for species that may be impacted but lack sufficient monitoring. This study aimed to investigate the relationship between arboreal mammals and climate variables and provide insights into the direct and indirect consequences of climate change on their survival. We systematically review the literature: (i) to identify gaps and biases in the current literature on the climate impact on arboreal mammals (ii) to identify observed (direct/indirect and positive/negative) climate change impacts of arboreal mammals (ii) to identify how different climate factors interact with each other and how various other pressures also interact to impact arboreal mammals, (iii) to identify how impacts vary among species or populations and across space and (iv) to explore main strategies addressed in the literature for managing and mitigating these climate change impacts and identify any issues with the suggested methods, along with ways they could be improved. Furthermore, we conducted a detailed bibliometric analysis of the studies from the systematic review to identify (i) annual trends in the publications, (ii) most relevant authors and sources, (iii) the contribution of corresponding authors by country, (iv) international collaboration networks, (v) co‐citation network analysis, and (vi) most cited global articles. We then performed a meta‐analysis to assess potential climate change driven distribution changes of arboreal mammals at three levels: taxonomic (family), geographic (continent), and conservation status (IUCN threat level).

## 2 Methodology

### 2.1 Review Protocol

This systematic review was conducted following the PRISMA 2020 guidelines (Preferred Reporting Items for Systematic Reviews and Meta‐Analyses) (Supplementary Material 1: Fig. 1) (Page et al. 2021). We conducted a systematic search on October 24, 2024, using the Scopus and Web of Science (WoS) online databases, and repeated the search on July 10, 2025, to capture any new studies. To improve the probability of identifying the relevant papers, a comprehensive search string was developed using diverse key terms related to climate change, temperature shifts, and extreme weather events, addressing both direct and indirect climate impacts on arboreal mammals. Seasonal studies were also included only if some climatic variables were measured (Supplementary Material 1: Inclusion exclusion criteria). The exact search strings were as follows:

Scopus:

TITLE‐ABS‐KEY((((mammal* OR species OR animal) AND (arboreal)) AND ((“climat* change” OR “global warming” OR “climat* warming” OR “environment* change” OR “changing climate” OR “Climate variability”) OR ((increase* OR decrease* OR shift*) near/5 AND temperature*) OR (weather AND (extreme OR severe)) OR (wildfire* OR cyclone* OR bushfire* OR heatwave* OR drought*))) AND NOT (terrestrial OR “ground dwelling” OR bird* OR reptil* OR amphibia* OR invertebrat*)) AND (LIMIT‐TO (LANGUAGE, “English”)) AND (LIMIT‐TO (DOCTYPE, “cp”) OR LIMIT‐TO (DOCTYPE, “ar”) OR LIMIT‐TO (DOCTYPE, “re”)).

Web of Science:

TS = (((mammal* OR species OR animal) AND (arboreal)) AND ((“climat* change” OR “global warming” OR “climat* warming” OR “environment* change” OR “changing climate” OR “climate variability”) OR ((increase* OR decrease* OR shift*) NEAR/5 temperature*) OR (weather AND (extreme OR severe)) OR (wildfire* OR cyclone* OR bushfire* OR heatwave* OR drought*))) NOT TS = ((terrestrial OR “ground dwelling” OR bird* OR reptil* OR amphibia* OR invertebrat*)) and Article or Review Article or Proceeding Paper (Document Types) and English (Languages).

We identified 747 studies from databases that matched the search terms. We used Covidence software to remove duplicates and to screen titles and abstracts (Veritas Health Innovation 2024). Following title and abstract screening, we retained 90 studies (see PRISMA and full review protocol, Supplementary Material 1). After reviewing the full texts, 59 papers were retained from the database searches. We further conducted backward and forward citation searching of each retained paper, as well as excluded review articles in the above step, and identified additional studies that met our inclusion criteria, resulting in a final total of 142 studies.

We extracted data from included papers, including the year of publication, study location (country and continent), species information (Scientific name as reported in the source study, and verified accepted scientific name with corresponding TSNs, taxonomic order, and family from ITIS), threat category (from the IUCN Red List, accessed 04 Dec 2025), climatic driver, study design, and findings (Supplementary Material 2). To determine whether arboreal mammal species that are threatened receive more research attention, we performed a Spearman's rank correlation. Species categorised as Data Deficient (DD) were excluded from threat status analysis due to insufficient information. A bibliometrics analysis of included papers was also performed using the “bibliometrix” package (Aria and Cuccurullo 2017). We conducted analyses using the R software version 4.2.2 (R core team 2022).

### 2.2 Meta Analysis

Following the protocol of Valdez et al. (2025), we used studies employing ecological niche and species distribution modelling (ENMs and SDMs) to predict future arboreal mammals' range changes in response to climate change to perform the meta‐analysis. The projected range changes ((future–present)/present), expressed as percentages under future climate scenarios, were used as the effect size. Sample size was defined as the number of records used in each study (Valdez et al. 2025) and was recorded where available. Standard deviations were extracted where available to represent the uncertainty associated with each effect size. However, many studies did not report standard deviations or confidence intervals directly for projected range change. In such cases, we used the best available quantitative uncertainty estimates reported by the original studies, including model‐level uncertainty measures such as the standard deviation of AUC across model replicates (Beale and Lennon 2012; Chi et al. 2023). In the absence of reported uncertainty estimates, standard deviations were estimated using values reported in comparable studies on the same or analogous species (Chi et al. 2023; Valdez et al. 2025). The included studies varied widely in the species examined, environmental predictors, and modelling approaches; therefore, we used random‐effects models to account for both within‐ and between‐study heterogeneity (Dettori et al. 2022). We fitted multilevel random‐effects meta‐analytic models using the *rma.mv* function in the *metafor* package (Viechtbauer 2010). The models included fixed effects for moderators (IUCN status, year of prediction, continent, and family of the species) and random effects for species and study ID to account for the non‐independence of multiple effect sizes (Cheung 2019). We treated the response variable as continuous and assumed it followed a normal distribution, using an identity link function to model it directly. We conducted analyses using the R software version 4.2.2 (R core team 2022).

## 3 Results and Discussion

Our review protocol identified 142 studies that described the impacts of climate factors on arboreal mammals (Supplementary Material 2). In this section, we (i) identify biases and gaps in the current literature, (ii) present insights from bibliometric analyses, (iii) explore climate drivers and their ecological relationships, (iv) examine observed impacts of climate change on arboreal mammals, (v) assess predicted range change effects using the meta‐analysis, (vi) review the mitigation strategies discussed in articles.

### 3.1 Knowledge Gaps and Biases in Climate Change Research on Arboreal Mammals

#### 3.1.1 Temporal Trends, Geographic Biases, and Taxonomic Gaps in Climate Research on Arboreal Mammals

**FIGURE 1:**
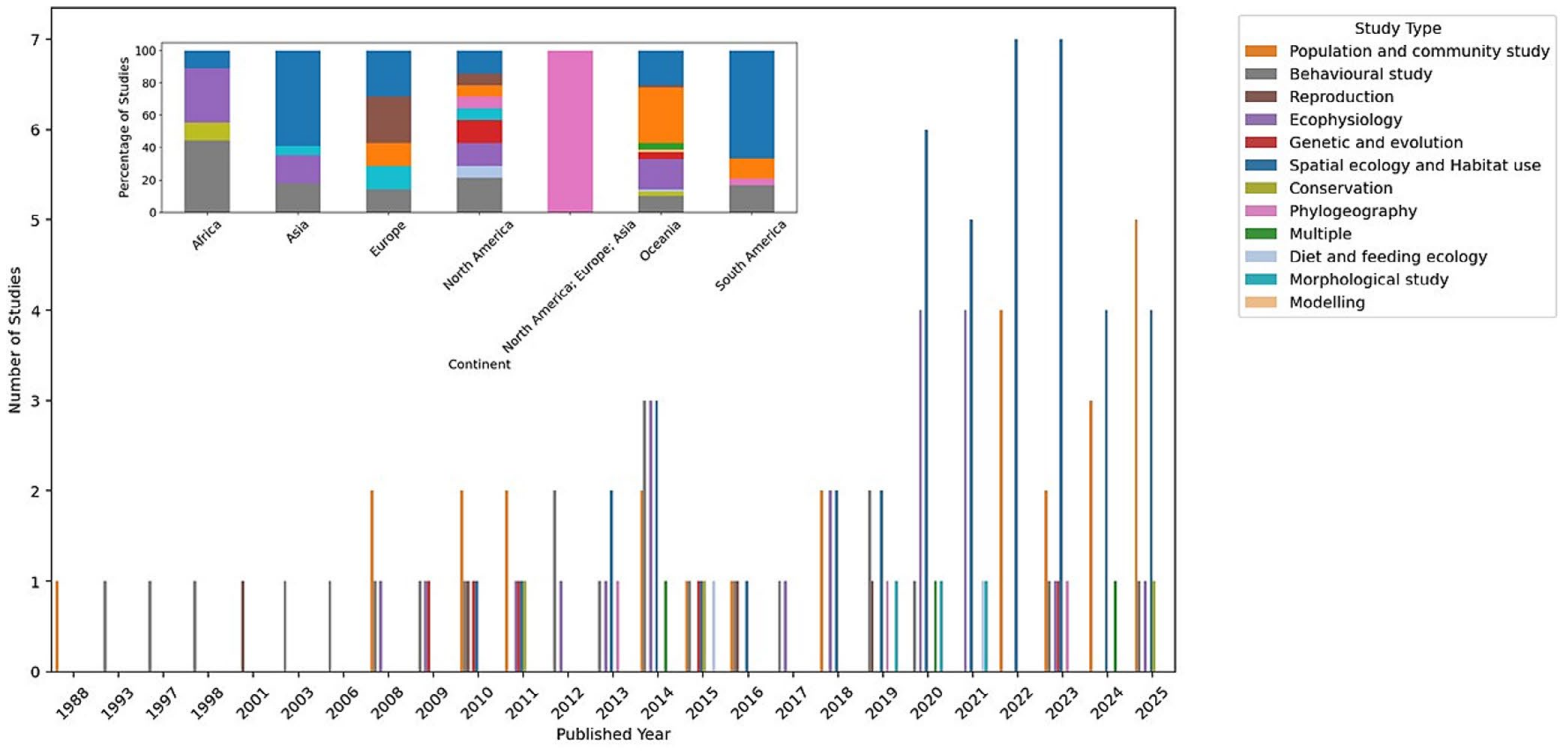
Number of studies published per year.

**FIGURE 2:**
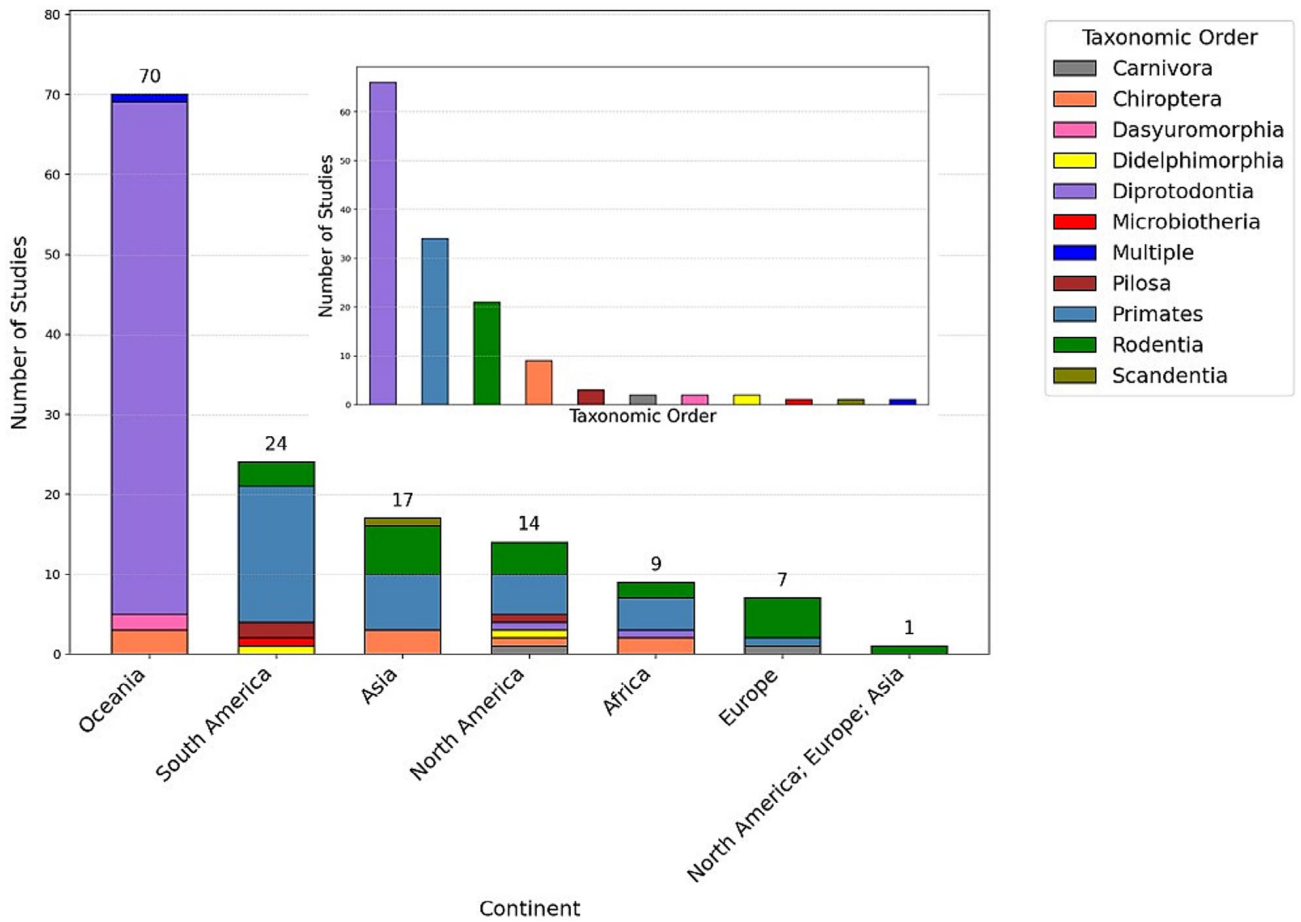
Number of studies published per continent and taxonomic order.

The literature reviewed included publications from 1988 to 2025, revealing a trend of an increasing number of publications over this period (Figure 1). The majority of the studies (49.3%) were conducted in Oceania (Figure 2), followed by South America (*n* = 24%) and Asia (17%), North America (14%), Africa (6.3%), and Europe (4.9%). One study focused on three continents (Asia, North America, and Europe). Notably, there was a regional bias, with most studies focused on Australia. This regional bias likely stems from Australia's high arboreal mammal diversity and their history of high mammal extinctions (Woinarski et al. 2015), with the first case directly linked to climate change (Fulton 2017). These events, coupled with recent climatic pressures (e.g., wildfires, droughts), have likely intensified research focus in Australia. In contrast, regions such as Europe and Africa may be underrepresented, likely due to their comparatively lower arboreal mammal diversity and endemism. The lack of information across most of Latin America (except Brazil) likely reflects both limited research effort given their high biodiversity in the region (Astiazarán‐Azcárraga et al. 2025) and the predominant use of camera traps, which restrict analyses to presence and activity patterns (Azcarraga et al. 2020). Our findings align with a study by Urban (2015), which identifies regions such as South America and Australia as among those facing the greatest risk of climate‐driven species extinctions.

A total of 45 studies investigated multiple species, whereas 97 studies focused on a single species. Most multi‐species studies focused on a single taxonomic order, with only one study covering multiple orders (Supplementary Material 2). *Diprotodontia* (a marsupial order including possums, koala, etc.) was the most frequently studied order (46.5%), followed by *Primates* (23.9%) and *Rodentia* (14.8%) (Figure 2). Also, there is a geographic bias in taxonomic coverage, where certain continents focus disproportionately on specific mammalian orders. For instance, in Australia, research mainly focuses on *Diprotodontia*, likely reflecting their regional uniqueness and conservation importance (Yang and Pavoine, 2023). Furthermore, studies from South America were primarily focused on primates, probably due to the region's diverse monkeys in tropical forests like the Amazon (Lynch 2017).

We examined a total of 281 species across all 142 studies (Supplementary Material 2). Koala ( *Phascolarctos cinereus* ) (*n* = 26) and greater glider ( *Petauroides Volans* ) (*n* = 19) were the most frequently studied arboreal mammal species, suggesting that these animals are major focal points of climate change studies. The koala has been recognised as one of the ten species most at risk from global climate change (Foden and Stuart 2009). However, the Spearman rank correlation test (*ρ* = 0.105, *p*‐value = 0.177) showed no association between how threatened a species is and how often it is studied, implying research effort was not influenced by the species' threat status.

Notably, many arboreal taxa (e.g., *Pilosa, Dasyuromorphia, Chiroptera*, and *Microbiotheria*) and regions (e.g., Europe and Africa) lack data, likely missing the full range of responses within the arboreal mammal group. We found that even the most studied arboreal species remain relatively understudied, given their wide distribution and ecological complexity. This highlights the need for increased research across both well‐studied and underrepresented taxa to address existing knowledge gaps. In addition, studies in regions such as Australia and South America remain insufficient to fully capture the impacts within these regions, given the immense diversity of arboreal mammals. Furthermore, longitudinal data are required to capture the long‐term effects of climate change on these species and ecosystems; therefore, conducting more research is essential to better understand and address these impacts over time. Consequently, arboreal mammals in under‐studied regions may be experiencing severe, undocumented impacts from climate change, a possibility that underscores the urgency of expanding research efforts. This is especially crucial for isolated or fragmented populations, which tend to face higher extinction risks due to reduced gene flow and limited adaptive capacity. Therefore, researchers should prioritize research on specific, taxonomically narrow topics rather than focusing solely on novelty or broad general interest. Such a shift would help reduce biases and encourage essential research efforts. Although studying every arboreal mammal is impractical, focusing on representative species across various habitats and regions can help identify general patterns and vulnerabilities.

Furthermore, such knowledge gaps and biases may also stem from broader disparities in research capacity, funding availability, and effectiveness of governance and policy implementation, as well as methodological constraints and taxonomic preferences favouring more accessible or charismatic species (Caldwell et al. 2024). Therefore, important gaps may remain, particularly in areas with limited research investment and monitoring capacity. Such research also requires consistent funding, proper data management, and collaborative efforts, all of which depend heavily on strong support from decision‐makers.

#### 3.1.2 Methodological Gaps and Biases

The predominant approach as classified in Rosalino et al. (2023), was based on spatial ecology and habitat use (32.8%), followed by population and community ecology (18.2%), ecophysiology (16.8%), behavioural (15.3%), genetic and evolution (3.6%), reproduction (2.9%), conservation (2.2%), morphology (2.2%), phylogeography (2.2%) and diet and feeding ecology (1.5%). Figure 1 illustrates the various approaches implemented across different continents, categorized by the taxonomic order of focal species. Australia had utilised different approaches mostly for the order *Diprotodontia*, while North America had carried out different methods mostly for *Rodentia*. Most ‘spatial ecology and habitat use’ studies (including shifts in distribution range) used SDMs, mainly MaxEnt (68%), followed by ensembles (22%). However, many of these studies have relied on limited or biased predictor sets, which can strongly affect model outcomes and inference (Breiman 2001). In contrast, modern machine‐learning and deep‐learning methods, which can handle large, high‐dimensional predictor sets (Hu et al. 2025; Steiner and Huettmann 2025), offer high predictive power and have the potential to provide more accurate insights into complex ecological relationships (Deneu et al. 2021), were rarely used in the studies reviewed. Moreover, few studies combined SDMs with other modelling approaches, such as connectivity models, which are key for capturing processes like dispersal and climate‐driven range shifts. Furthermore, mechanistic studies, which examine life‐history and functional traits (e.g., reproduction, survival), were rare, yet are vital for understanding species' adaptive capacity to climate change. However, no studies assessed climate change impacts on arboreal mammals in relation to disease or pathogens. Furthermore, although seasonal studies often classify climate using broad wet and dry seasons, incorporating actual ambient temperature measurements and annual temperature ranges would provide a more detailed understanding of mammalian responses to climate variability.

#### 3.1.3 Weather and Climate Variables Presentation

The climate drivers represented in most of the reviewed studies (89%) were either directly (e.g., high temperature) or indirectly (e.g., droughts, wildfire) linked to temperature, with elevated temperatures emerging as the primary factor influencing climate change impacts on most arboreal mammals. Within the reviewed articles (excluding predictive studies), there were only a few studies (about 5%) that specifically addressed severe weather events such as cyclones and storms (Kanowski et al. 2008; Nowack et al. 2015; Wilson et al. 2008), likely due to their unpredictable nature and the difficulty of collecting data during and after such events. Only 38% of studies considered multiple predictors; most focused on a single factor, potentially missing complex combined or interacting effects of climate factors.

#### 3.1.4 Relationships Identification

The relationship between the country, the family, and the climate driver of included studies is visualized using a Sankey diagram, where the flow line colour represents a specific country (Supplementary Material 1: Fig. 3). Here, we excluded all the predictive studies, considering only empirical studies to ensure a more grounded understanding of observed patterns and real‐world data. To illustrate the predictive and empirical impacts in relation to country, climate driver, and family, an additional Sankey diagram is provided in Supplementary Material 1: (Fig. 4). Most studies were focused on *Pseudocheiridae, Atelidae*, and *Phalangeridae* families, indicating that species within these groups are particularly affected by climate change. Australia had experienced several climate events (e.g., cyclones, storms, droughts), prompting numerous individual studies across different families, mainly within the order *Diprotodontia*, such as *Pseudocheiridae, Phalangeridae*, and *Dasuridae*. Temperature was the most significant climate driver affecting numerous arboreal mammals worldwide.

### 3.2 Bibliometric Analysis

All graphs and tables relevant to the Bibliometric analysis of included papers are provided in Supplementary Material 1. Table 1 shows the top‐cited studies. The analysis of international collaborations across studies reveals that Australia is the most frequent partner, involved in ten collaborations, followed by Brazil (seven) and Canada (three). Notably, Australia plays a central role in cross‐national research efforts, particularly with partners such as Germany, the Netherlands, and the USA. The citation analysis reveals that Australia leads with the highest total citations (1845), followed by the United Kingdom (358) and the USA (355). Brazil and New Zealand also show notable contributions, with 221 and 204 citations, respectively.

**TABLE 1:** Top‐cited studies.

Author (year)	Source	Total citations	Avg. citations/year
Kearney et al. (2010)	CONSERV LETT	406	25.38
Welbergen et al. (2008)	Proc R Soc B	358	19.89
Sedgeley (2001)	J APPL ECOL	221	8.94

### 3.3 Climate Change Impacts on Arboreal Mammals

We identified both direct and indirect impacts on arboreal mammals from climate change, ranging from immediate threats like heat stress and habitat loss to broader ecological effects. We classified those observed impacts into categories as shown in Table 2. A complete record of observed and predicted impacts is provided in Supplementary Material 2. In this section, we outline six key questions that will guide our understanding of current knowledge on climate change impacts on arboreal mammals.

**TABLE 2:** Summary of reported climate impacts on arboreal mammals across studies.

Species	Climate factor	Impact	References
*Demographic changes*			
Koala	Drought	80% population decline, local extinction	(Lunney et al. 2014; Seabrook et al. 2011)
Yellow‐bellied glider	Drought Wildfire	~48% decline after drought; ~26% decline after wildfires	(Bilney et al. 2022; Goldingay et al. 2023)
Greater glider	Warm nights (> 20°C)	Negative population impacts	(Wagner et al. 2020)
	Wildfire	Low effective population sizes, increased local extinction risk, Lower abundance, and slow colonization at severely burnt sites	(Chia et al. 2015; May‐Stubbles et al. 2022)
	Drought	Consistently low detections indicating a decline	(Goldingay et al. 2022)
	High temperatures	Reduced the likelihood of occurrence	(Smith and Smith 2018)
Capuchins, Spider monkeys	Drought	Infant mortality spiked, reproduction halted	(Campos et al. 2020)
Little Brown Myotis	High temperature	Reproduction failed	(Adams 2010)
Siberian flying squirrel	Rising winter temperatures	The population declines over 15 years	(Koskimäki et al. 2014)
Ringtail possums	Heat waves	Population declines	(de la Fuente and Williams 2023)
Siberian flying squirrel	Increased winter precipitation	Increase population	(Selonen et al. 2019)
*Behavioural changes*			
Koala	High temperatures	Selected shelter trees, particularly taller trees at lower elevations, tree‐hugging, more visits to water sources, and drinking more water, however, were ineffective in moderating body temperature	(Briscoe et al. 2014; Crowther et al. 2014; Mella et al. 2019, 2020, 2024; Smith et al. 2013)
	Rainfall	Licked water running down tree trunks, resumed feeding shortly after rain events	(Mella et al. 2020)
	Drought	Selected riparian habitats, shifted diet to higher leaf‐moisture content species to cope with water scarcity	(Davies et al. 2014; Seabrook et al. 2011; Smith et al. 2013)
New Zealand Long‐tailed Bat, Prince Bernhard's titi monkey, Prince Bernhard's titi monkey, Mantled howling monkey, Common brushtail possum, Lesser dog‐faced fruit bat	Temperature extremes	Selected cooler microhabitats to buffer from extreme temperatures	(Isaac et al. 2008; Lopes and Bicca‐Marques 2017; Sedgeley 2001; Thompson et al. 2016; Welman et al. 2017)
Black‐and‐gold howler monkey	Higher temperatures	Adjusted resting sites and postures for temperature regulation	(Bicca‐Marques and Calegaro‐Marques 1998; Brividoro et al. 2023; Wark et al. 2014; Zalewski 1997)
Pine marten			
Colobus monkey			
Chimpanzee			(Kosheleff and Anderson 2009)
Prince Bernhard's titi monkey			(Lopes and Bicca‐Marques 2017)
Greater glider	High temperatures	Exited nest boxes	(Gracanin et al. 2025)
Brown‐throated sloth	High temperatures	Increased food intake at higher temperatures to facilitate digestion and energy acquisition	(Cliffe et al. 2015)
Bonin flying fox	High temperatures	Clustering behaviour to manage thermoregulatory challenges and conserve energy	(Rhind 2003)
Brush‐tailed phascogale	High temperatures	Nest sharing to conserve energy and manage heat stress	(Sugita and Ueda 2013)
Japanese dormouse	Rainfall	Reduced activity during rainfall events	(Giné et al. 2012)
Indian giant flying squirrel			(Kuo and Lee 2012)
Thin‐spined porcupine	Rainfall	Shortened feeding time	(Suzuki and Ando 2019)
Sugar glider	Storm	Reduced activity and foraging, entered deep torpor	(Nowack et al. 2018)
Greater glider	Wildfire	Home range fidelity	(Miritis et al. 2025)
*Physiological responses*			
Lemurs	Rising temperatures during hibernation	Hyperthermia due to increased metabolic rates driven by the Arrhenius effect	(Lovegrove et al. 2014)
Flying foxes	Extreme heat	Mass die‐offs exceeding 30,000 individuals (1994–2007); 3500 deaths in a 2002 heatwave	(Welbergen et al. 2008)
Koala	Heat and drought	Exhibits heterothermy (32.4°C–40.8°C), Increased physiological stress, 63% of the population died	(Davies et al. 2014; Gordon et al. 1988; Mella et al. 2024)
Green ringtail possum	High temperatures (> 30°C)	Stores heat, but the strategy is ineffective for prolonged periods	(Krockenberger et al. 2012)
Common ringtail possum	Extreme heat (29°C–39°C)	Facultative hyperthermia delays dehydration, but > 35°C–36°C triggers active cooling	(Knight 2020; Turner 2020)
Commerson's Leaf‐nosed Bat	Heat stress	Torpor + hyperthermia	(Reher et al. 2022)
Tropical free‐ranging bats	Warm conditions	Flexible heterothermy/torpor	(Stawski and Geiser 2012)
Lesser dog‐faced fruit bat	Extreme heat (> 31°C)	Highly vulnerable to hyperthermia	(Welman et al. 2018)
*Distributional shift*			
Southern flying squirrel	Warmer winters	Northward range shift in Maine over 18 years	(Wood et al. 2016)
Greater glider	Higher temperatures	More detections at higher elevations	(Wagner et al. 2020)
Habromys spp.	Glacial–interglacial cycles	Distributional shifts	(León‐Paniagua and Guevara 2019)
Siberian flying squirrel	Rising winter temperatures	Range expansion, and Hybridization	(Garroway et al. 2010)
Koala	Drought	12% decrease in suitable habitat	(Kotzur et al. 2024)
*Resource limitation*			
Koala	Drought	85% of koala mortalities were associated with browse moisture falling under 51% fresh weight	(Clifton 2010)
*Genetic/evolutionary impact*			
Siberian flying squirrel	Rising winter temperatures	Rapid genetic diversity reduction, despite high gene flow	(Garroway et al. 2010)
Habromys spp.	Glacial–interglacial cycles	Genetic differentiation	(León‐Paniagua and Guevara 2019)
*Phenological changes*			
Siberian flying squirrel	Warmer winters, high precipitation	Adjusted reproductive cycle to earlier reproduction	(Selonen et al. 2019; Selonen et al. 2016)

#### 3.3.1 What Are the Observed Climate Change Impacts on Arboreal Mammals in the Current Literature? Are They Positive, Negative or Neutral?

Findings from the reviewed studies demonstrated that climate extremes negatively affect many arboreal mammals in several ways (Table 2). These impacts include direct physiological stress (e.g., hyperthermia, elevated cortisol levels, metabolic disruption, mass mortality events; (Davies et al. 2014; Lovegrove et al. 2014; Mella et al. 2024; Turner 2020; Welbergen et al. 2008)), demographic declines (e.g., reduced detection rates, population collapse, and local extinctions; (Bilney et al. 2022; Goldingay et al. 2023; Knipler et al. 2023; Wagner et al. 2020), loss of genetic diversity (Knipler et al. 2023; León‐Paniagua and Guevara 2019)) and distributional shifts (e.g., range contractions and elevation shifts; (Wagner et al. 2020; Wood et al. 2016)). In addition, severe weather events such as wildfires have resulted in long‐term declines in sensitive species such as yellow‐bellied gliders and greater gliders (Bilney et al. 2022). Furthermore, behavioural responses altering foraging efficiency and energy balance have also been recorded (Isaac et al. 2008; Mella et al. 2020) along with reproductive adjustments (Selonen et al. 2019; Selonen et al. 2016). Alterations in precipitation patterns had an influence on water‐seeking behaviour, dietary shifts, and activity patterns of some arboreal mammals (Davies et al. 2014; Giné et al. 2012; Suzuki and Ando 2019). Furthermore, shifts in climate regimes are anticipated to affect habitat quality and resource availability, indirectly impacting arboreal species that rely on complex vertical structures (e.g., tree hollows (Banks et al. 2011)) and foliage for survival and reproduction.

Although most impacts were negative (over 90%), some studies reported positive effects. One positive impact noted was genetic adaptation in certain populations (Banks et al. 2015; Garroway et al. 2011; Knipler et al. 2023) of arboreal mammals, suggesting potential for evolutionary resilience under climate change. Siberian flying squirrel populations increased in response to higher levels of winter precipitation (Selonen et al. 2019).

In a few cases, climate change showed no observable effects. Some marsupials were found to be resilient, with no catastrophic loss of habitat or population after major cyclones (Kanowski et al. 2008). Mountain brushtail possums in mesic refuges maintained high survival rates regardless of rainfall, suggesting fine‐scale refugia mitigate climate effects (Banks et al. 2015). Phascogale and sugar gliders tolerated high nest temperatures with no mortality or physiological stress (Rowland et al. 2017). However, findings of ‘no effect’ do not imply an absence of risk, and may instead reflect species' adaptive capacity, phenotypic plasticity, or limitations in the temporal scale or sensitivity of the methods used to detect change. Thus, the interpretation of such findings should be approached with caution.

#### 3.3.2 How Does Climate Change Directly Impact the Physiological Functioning of Arboreal Mammals?

Among the studies reviewed, there were few articles addressing direct physiological impacts on arboreal mammals (Gordon et al. 1988; Lovegrove et al. 2014; Mella et al. 2024; Mella et al. 2020; Mella et al. 2019; Turner 2020; Welbergen et al. 2008; Welman et al. 2017). Thermal tolerances were found to be species‐specific; for instance, koalas can tolerate temperatures from 32.4°C to 40.8°C (Mella et al. 2024), possums need cooling above 35°C–36°C (Turner 2020), and the lesser dog‐faced fruit bat struggles with temperatures over 31°C (Welman et al. 2018). However, studies show that prolonged heat exposure can surpass the adaptive limits of arboreal mammals (e.g., flying foxes, koalas, greater gliders, howler monkeys and yellow‐bellied gliders), resulting in elevated physiological stress and increased risk of mortality (Goldingay et al. 2023; Gordon et al. 1988; Pozo‐Montuy et al. 2024; Welbergen et al. 2008), thereby contributing to population declines. In contrast, no studies were found that reported population declines directly attributable to decreased temperatures. This suggests that heat stress may play an important role in population declines in arboreal mammals.

#### 3.3.3 How Do Different Climate Factors Interact to Affect Arboreal Mammals? (Synergistic and Antagonistic Effects of Multiple Drivers)

The impacts of climate change on arboreal mammals can be best understood by examining how multiple climatic variables interact, rather than focusing on single factors in isolation. Various climatic variables can exert contrasting effects; for example, rising temperature might be beneficial to species in one aspect, but detrimental to the species in another aspect. For instance, in this systematic review, we found a study on the Siberian flying squirrel in which rising winter temperatures negatively affected populations (Koskimäki et al. 2014), while in another study, increased winter precipitation had a positive effect (Selonen et al. 2016). Another instance is that precipitation negatively affected the activities of the Indian giant squirrel, whereas temperature had no significant impact (Kuo and Lee 2012). Therefore, in the above cases, rising temperatures might have one effect on those species, but when combined with increased precipitation, the overall impact might be very different. Because of these interacting and sometimes opposing effects, it becomes difficult for researchers to draw definitive conclusions when examining the influence of a single variable in isolation. Furthermore, drought is a prime example of a synergistic effect of climate factors through the interaction of temperature and low precipitation. These events not only directly affect species (e.g., via physiological stress) but also reduce the availability of essential food and water sources (Goldingay et al. 2023). These interactions highlight the need for a more nuanced understanding of how multiple stressors operate simultaneously in natural environments and how these interactions must be integrated into conservation strategies of arboreal mammals.

#### 3.3.4 What Ecological, Structural, Governance and Anthropogenic Factors Indirectly Affect Arboreal Mammals Under Climate Change, and Are All Arboreal Mammals Affected in the Same Way by These Factors?

Understanding arboreal mammals and climate change goes beyond direct effects, as they face unique challenges due to their strong dependence on forests for survival. Unlike ground‐dwelling mammals, many of these species are reluctant to move across open ground because they are evolutionarily adapted to life in the treetops, not the forest floor (Dublin 1903; Harel et al. 2022). Their movement, foraging strategies, locating mates, and predator avoidance are built around tree‐to‐tree travel, making exposure on the ground risky, energetically costly, and outside their natural behaviour (Harel et al. 2022; Makin et al. 2012). For this reason, many arboreal mammals have evolved specialised adaptations such as prehensile or semi‐prehensile tails, gliding membranes, friction‐enhancing foot pads, and enhanced spatial memory to judge branch stability and gap distances (Byrnes and Spence 2011; Cartmill 2017; Poindexter et al. 2023). However, this also makes them disproportionately sensitive to canopy and tree disruption. As climate envelopes (the specific climate conditions where species and ecosystems can thrive) shift in response to changing temperature and rainfall patterns, the suitable habitat for these species is also shifting across the landscape (Littlefield et al. 2017). Yet because many arboreal mammals move primarily through continuous treetops, even small gaps between forest patches can prevent them from tracking these shifting environments, increasing the risk of isolation, range contraction, and population decline (Whitworth et al. 2019). Moreover, climate‐induced shifts in tree distributions and fruiting/flowering cycles can create mismatches between resource availability and their ecological requirements (Mella et al. 2019; Selonen et al. 2020; Selonen et al. 2016). Studies have shown that changes in temperature and rainfall alter food availability and leaf moisture, imposing nutritional stress and seasonal food shortages that may result in lower reproductive success and higher mortality in some arboreal mammals (Adams 2010; Clifton 2010; Gordon et al. 1988; Selonen et al. 2016).

However, climate change rarely acts alone. Instead, it amplifies other major pressures, such as habitat fragmentation, more frequent wildfires, prolonged droughts, and overall forest degradation (Abram et al. 2021; Nitschke and Innes 2006; Opdam and Wascher 2004). Due to this interactive and broad‐scale effect, climate change may sometimes be more important than other drivers (Nitschke et al. 2020; Nitschke and Innes 2006). These pressures accelerate the loss of old‐growth forests, shifting them toward early‐successional stages. To assess such shifts, canopy condition is often measured using metrics such as canopy height, leaf area index, and foliage/canopy density metrics that reflect successional stage and structural complexity (Smith et al. 2008). As forests lose large trees and mature canopy layers, key resources for arboreal mammals, such as tree hollows (Banks et al. 2011; Isaac et al. 2008), tall canopies (Palminteri et al. 2012), microclimatic buffering (Adam et al. 2025; Lindo and Winchester 2013), and safe movement pathways (Davies et al. 2013; Lane et al. 2024; McLean et al. 2016), become scarce. Thinner, patchier canopies hinder their movements, expose arboreal mammals to extreme temperatures and predators, and reduce access to high‐quality food, which ultimately reduces both their abundance and diversity (Adam et al. 2025; Isaac et al. 2008; Lindo and Winchester 2013; Reher et al. 2022; Sedgeley 2001; van Jaarsveld et al. 2021). In this way, for arboreal mammals, canopy condition and forest quality largely determine whether they persist, adapt, or decline under ongoing warming and disturbance (Cally et al. 2025; Cudney‐Valenzuela et al. 2023; Lindenmayer et al. 2024). Thus, vulnerability in arboreal mammals stems not only from climatic interactions themselves, but from other pressures that climate change intensifies. However, these effects may occur over different time scales; for instance, interaction between climate factors might happen more quickly, while fragmentation or forest degradation unfolds slowly, requiring different management interventions.

These Indirect ecological impacts affect arboreal mammals differently, depending on their canopy dependence and the specificity of their diet or movement behaviour. Highly canopy‐bound mammals that do not fly (e.g., lemuroid ringtail possum, howler monkeys, sloths and greater gliders) are most vulnerable because they rely on continuous canopy and rarely descend to the ground, leaving them little flexibility (Wilson et al. 2007). In contrast, mammals that also spend part of their life on the ground, such as some squirrels and tree kangaroos, tend to be more adaptable because they can move across short breaks in canopy cover and exploit resources across different forest layers. Furthermore, species with narrow diets are more vulnerable than generalist feeders because they struggle to switch to alternative foods when their preferred resources decline (Colles et al. 2009; Kotzur et al. 2024; Reed and Tosh 2019). Nonetheless, all arboreal mammals remain highly vulnerable to the combined pressures, all of which may drive them to use the ground more frequently, increasing their susceptibility to risks (Eppley et al. 2022).

Anthropogenic climate change has emerged as a pressing global challenge (Peng et al. 2026). Despite decades of international climate agreements and conservation initiatives, global GHG emissions continue to rise, highlighting persistent limitations in environmental governance, mitigation effectiveness, and policy implementation (IEA 2026). In many regions, economic priorities associated with resource extraction (e.g., timber extraction), commercial agriculture (e.g., livestock and soy and oil palm cultivation), and land conversion continue to outweigh biodiversity conservation objectives (Huettmann and Young 2022), intensifying pressures on arboreal ecosystems through habitat degradation, fragmentation, and climate instability. Moreover, forest management practices implemented under the guise of sustainable forest management or carbon‐sequestration initiatives may still contribute to increased logging and the replacement of ecologically complex old‐growth forests with plantations (Huettmann and Young 2022), potentially reducing habitat quality for many arboreal mammals. These institutional and policy failures often magnify the ecological impacts described above, limiting conservation effectiveness and reducing biodiversity (Cudney‐Valenzuela et al. 2023).

#### 3.3.5 How Do Impacts Vary in Their Responses Across Species, Populations, and Space?

Emerging studies highlight that species‐specific traits and needs play a crucial role in shaping responses and vulnerabilities to climate change of various species (Germain et al. 2023; Viza et al. 2023; Zografou et al. 2021). Supporting this, we found that even arboreal mammals belonging to the same order sharing the same environment can exhibit markedly different responses to climate impacts. While long‐term monitoring in eastern Australia showed minimal drought effects on koalas, greater gliders in the same landscapes showed consistent declines (Goldingay et al. 2022). Similarly, in a long‐term study on six arboreal marsupials, only greater gliders showed a significant decline linked to climate, with lower occurrence in hotter areas (Lindenmayer et al. 2024). These examples suggest a variation in climate sensitivity, possibly due to differences in thermal tolerances or specific resource needs (e.g., tree hollows). Sometimes populations of the same species responded differently to climate change, with variation arising from genetic differences, developmental history, or age‐related declines in physiological capacity (Aujard et al. 2006; Noakes et al. 2021; Reher et al. 2022). Sometimes responses of the same species varied spatially, where impacts were evident at some sites but not others (Lindenmayer et al. 2024), reflecting differences in local environments and conditions. These patterns of variation, between species, among populations, and even within a single population, highlight that the climate change impacts on arboreal mammals are complex and context‐dependent.

#### 3.3.6 What Are the Potential Climate Change Effects of Arboreal Mammals? Insights From the Meta‐Analysis

**FIGURE 3:**
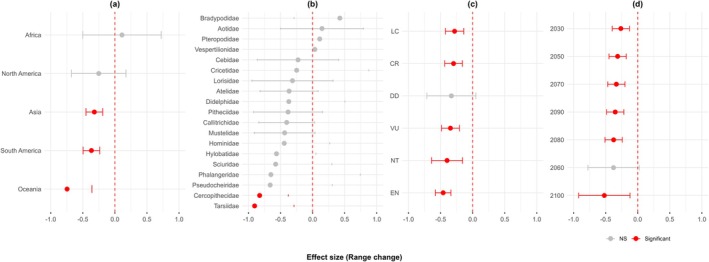
Mean predicted range change under climate change for each (a) continent, (b) family, (c) IUCN status, (d) year of prediction.

Potential climate change impacts using future climate projections on arboreal mammals were commonly investigated using SDMs. Most of the projected climate change impacts are negative, with significant range and habitat loss (Supplementary Material 2) posing a serious threat to arboreal mammal diversity. Multiple arboreal mammals within the same order in the same landscape can be expected to show different responses to climate change in predictive studies, similar to what was observed in empirical studies (da Silva et al. 2022; Sales et al. 2020) (Section 3.3.5). Out of the 142 articles identified in the systematic review, 28 met the criteria for inclusion. Our meta‐analysis included 189 species and 473 effect sizes. The studies in this dataset (Supplementary Material 2) were distributed across South America (43.8%), Asia (37.5%), Oceania (9.38%), North America (6.25%), and Africa (3.12%). Among the families included, *Callitrichidae* accounted for 19.5%, *Pitheciidae* 17.9%, *Cercopithecidae* 13.7% and *Atelidae* 11.1%. Our analysis detected substantial heterogeneity across studies, with the predictive variables accounting for a significant proportion of the variability in effect sizes (QM = 5.15 × 10^7^, *p* < 0.0001). This indicates that the potential range shifts vary considerably among the species examined in this study. The overall mean effect size across species was around −0.50 (SE = 0.041, *z* = −12.30, *p* < 0.001), indicating a strong and significant negative predicted change in mammals' future ranges. Several families, including *Tarsiidae, Cercopithecidae*, *Callitrichidae*, and *Pseudocheiridae*, showed significant future range contractions. Predicted range changes were generally negative across continents and IUCN threat categories, but none were statistically significant. Furthermore, our meta‐analysis revealed a progressive and statistically significant contraction of species ranges projected from 2030 to 2100 (Figure 3).

### 3.4 Managing and Mitigating the Effects of Climate Change on Arboreal Mammals

This section aims to summarise the main mitigation strategies proposed in the current literature to address these negative impacts, highlight any issues with the suggested methods, and provide how those methods can be improved.

#### 3.4.1 Identification of Climate Refugia and Thermally Buffered Habitats

In Section 3.3, we identified increasing temperature as the primary driver of the negative impacts on arboreal mammals. Given that, many studies in the analysis emphasize the importance of protecting thermally buffered habitats and establishing thermal refugia to combat climate change's impacts on them. Microhabitats play a critical role in buffering animals from climate extremes (Krockenberger et al. 2012; Scheffers et al. 2014; van Jaarsveld et al. 2021). Protecting cooler, higher‐elevated areas is also important, as these act as climatic refugia for several arboreal mammals (Crowther et al. 2014; Krockenberger et al. 2012; Lindenmayer et al. 2022; Luo et al. 2015; Wagner et al. 2020). Similarly, some studies emphasize the importance of identifying drought refuges for arboreal mammals (Gallahar et al. 2021; Goldingay et al. 2023; Kotzur et al. 2024; May‐Stubbles et al. 2022). The next challenge is determining how to identify these refugia and assess their effectiveness. Identifying refugia for arboreal mammals requires a multi‐faceted approach, utilizing both technological and ecological methods. Remote sensing data (e.g., satellite or aerial data; (Kotzur et al. 2024)) can help identify areas that remain relatively stable in the face of climate change. Furthermore, species distribution models and microclimate models can be used to predict areas with suitable environmental conditions for species to thrive in the future under climate change (da Silva et al. 2022; Koli et al. 2023; Luo et al. 2015). Additionally, field surveys and regular monitoring are crucial to validate these predictions and assess the actual use of potential refugial zones by the species (Banks et al. 2015). Genetic studies may also offer insights into areas where species are more resilient to climate stressors (Knipler et al. 2023). Finally, fire and drought risk maps can further refine our understanding of which areas will serve as refugia (Meddens et al. 2018; Piccioli Cappelli et al. 2021).

The studies we reviewed showed that for some arboreal marsupials (e.g., common bushtail possums, common ringtail possums, and sugar gliders), tree hollows provide essential thermal refuges (Rowland et al. 2017). In contrast, artificial nest boxes currently fail to provide adequate thermal regulation during extreme summer temperatures for some vulnerable species (e.g., greater glider) (Rowland et al. 2017), while others, such as the sugar gliders, may tolerate the conditions provided by these boxes (Goldingay 2015; Goldingay and Thomas 2021). Improving nest‐box designs to better mimic the thermal conditions of natural hollows has been highly recommended by studies (Callan et al. 2023; Larson et al. 2018; Rowland et al. 2017). However, it is also important to carefully evaluate their broader ecological impacts. Nest boxes can sometimes attract predators (Goldingay et al. 2020; McComb et al. 2019) or overheat in extreme temperatures or alter species behaviour in unintended ways (e.g., increase competition, natural nesting behaviours), potentially undermining conservation objectives. Future conservation efforts should weigh these risks and explore design modifications, such as improved insulation, predator guards, and strategic placement, to enhance their effectiveness while minimizing potential perverse effects.

#### 3.4.2 Habitat Protection, Restoration, and Management

As discussed in Section 3.3.4, arboreal mammals face distinct challenges primarily due to their dependence on forested habitats for survival, highlighting that conservation efforts must extend beyond habitat retention alone. As climate change alters forests, maintaining and enhancing connectivity between fragmented areas is also crucial for arboreal mammals (de Oliveira et al. 2023). Controlling deforestation and promoting forest regeneration are key strategies for mitigating the effects of climate change across diverse arboreal taxa such as sloths (Santos et al. 2022; Tourinho et al. 2022), primates (Luo et al. 2015; Sales et al. 2020), and marsupials (Crowther et al. 2014). Research emphasizes that protecting forests with complex structures such as abundant tree hollows (Isaac et al. 2008) or mature trees (Crowther et al. 2014; Kuo and Lee 2012) is essential for some species, while poor quality habitat exacerbates the impact of climate extremes (Cally et al. 2025; Seabrook et al. 2014). Therefore, what matters most for these species is not merely the presence of trees, but the presence of old‐growth trees. Therefore, conservation efforts should focus on protecting existing old‐growth forests from being cleared, degraded, or converted into production forests. As climate change advances, the spatial distribution of suitable habitats is projected to shift, and these arboreal mammals may be forced to track shifting climate envelopes more rapidly than in the past. Given their heightened sensitivity to canopy disruption and habitat fragmentation and the role of climate change in amplifying these disturbances (as discussed in Section 3.3.4), enhancing canopy connectivity is both a conservation priority and an essential adaptation strategy (Crowther et al. 2014; Littlefield et al. 2019; Soanes and van der Ree 2015; Taylor et al. 1993). Increasing connectivity is the most consistently recommended adaptation strategy for conserving biodiversity under climate change (Heller and Zavaleta 2009). At local scales, structures such as artificial canopy bridges can be recommended to enhance connectivity (Nekaris et al. 2021). However, careful attention is needed to determine where these bridges should be placed. At larger spatial scales, connectivity models could be used to identify appropriate climate‐resilient corridors incorporating multiple species; however, our review could only identify one such model (Zhang et al. 2019). This may be due to a lack of data due to difficulty in monitoring arboreal mammals in complex forest canopies (Kays and Allison 2001). Therefore, there is an opportunity for future landscape management strategies to focus on integrating climate change‐driven habitat connectivity to support arboreal mammals' range shifts. Here, the role of private lands is also essential, as they can serve as refuges or corridors for species (Ivanova and Cook 2020). It is predicted that increasing protected areas by 2%–10% can significantly reduce the extinction risk for primates, particularly in the Northeastern Amazon (da Silva et al. 2022). The conservation of non‐protected areas adjacent to reserves is also crucial for sustaining endemic species, which rely on specific habitats (Luo et al. 2015; Molloy et al. 2014; Shameer et al. 2023). While protected areas provide vital refuges, these habitats alone may not be enough to ensure long‐term population viability. As such, surrounding areas, even within developed land, play a key role in providing additional resources, corridors, buffer zones, and refuges (Gallahar et al. 2021). Finally, wildfires and droughts together cause severe loss of habitat and resources for arboreal mammals. Therefore, effective conservation efforts must incorporate strategies for managing both fire and drought risks.

Addressing conservation challenges within the Anthropocene may also require recognising the limitations of conventional forestry and conservation approaches including overreliance on protected areas alone and forest management systems that prioritise resource use and production over long‐term ecological resilience and biodiversity conservation (Barnes et al. 2018; Ellis 2019; Huettmann and Young 2022). Future strategies may therefore benefit from more integrated frameworks that emphasise human–wildlife coexistence, sustainable land management, landscape connectivity, and biodiversity conservation alongside agriculture, forestry, urban development, and broader governance systems (Ellis 2019). In this context, Indigenous knowledge systems and community‐based stewardship may contribute valuable insights (Bardsley and Wiseman 2016). This may help balance ecological, social, and economic needs while promoting more integrated and sustainable approaches to conservation under climate change, consistent with broader goals such as the Sustainable Development Goals (SDGs) (Ellis 2019).

#### 3.4.3 Species‐Specific Conservation Actions

As discussed in Section 3.3, climate change impacts were species‐specific, and some mammals are more vulnerable than others. Therefore, studies emphasize the importance of conducting more species‐specific research because understanding unique ecological needs, behaviours, and vulnerabilities of individual species is crucial for developing effective conservation strategies. For instance, Welbergen et al. (2008), recommend close monitoring of flying fox colonies in areas where temperatures are forecast to exceed 42.0°C. In addition, regular population censuses of targeted species may be essential to detect changes in population size over time and to better understand climate‐driven population trends. Several studies highlight the need to combine physiological assessments with ecological studies to identify specific stressors and the resources needed for long‐term survival of these species in response to climate change (Davies et al. 2014; Meyer et al. 2014; Nespolo et al. 2024; Wark et al. 2014). Cui et al. (2020) suggests prioritizing life‐history adaptations and functional traits over traditional ecogeographical rules for predicting species' climate change vulnerability. Some arboreal mammals may exhibit potential for adaptation to climate change due to genetic variation, and those studies emphasize further management of those traits, as these genetic adaptations alone may not suffice without proper management (Cui et al. 2020; Knipler et al. 2023). Therefore, there is a growing need for species‐specific research to better understand the unique vulnerabilities and adaptive capacities of arboreal mammals in the face of climate change. While important, focusing research solely on individual species has the potential to overlook important ecosystem‐wide interactions, especially when species are interdependent or share habitats (Hagen et al. 2012). Also, prioritizing one species may inadvertently harm others that rely on the same resources or habitats, or disrupt critical ecological processes (Faria et al. 2023). Despite these drawbacks, the need for species‐specific research and conservation actions is essential to address the increasingly complex threats posed by climate change, especially for vulnerable species. Balancing this approach with broader conservation strategies (e.g., incorporating closely related multiple species) while maintaining ecosystem resilience is key to ensuring the survival of arboreal mammals in the face of climate change.

### 3.5 Limitations and Recommendations for Future Reviews

This study comprehensively evaluates the impacts of climate change on arboreal mammals through a systematic review, meta‐analysis, and a bibliometric analysis. However, several limitations exist in this research, as with any systematic review.

Due to the exclusion of grey literature, unpublished studies, and non‐English literature, some relevant studies may have been missed, which may lead to undetected effects and may contribute to increased geographic and taxonomic biases, potentially limiting the ability to fully capture the impacts on arboreal mammals. Furthermore, expanded search strategies (e.g., including specific genus‐ and family‐level terms) and including several databases may help improve coverage and yield more comprehensive results. In addition, meta‐analysis focused on ENMs and SDMs for predicted range change is subject to inherent uncertainties and assumptions associated with these modelling approaches (Elith and Leathwick 2009), and results should therefore be interpreted with caution. For instance, key ecological processes such as species interactions, evolutionary adaptation, dispersal limitations, and habitat change are often not explicitly represented in these models (Buckley et al. 2010). The omission of these processes may influence predicted range changes and affect predicted risks in either direction. A further limitation of this meta‐analysis is that uncertainty estimates were not reported consistently across the primary studies. Because many studies did not provide SDs or confidence intervals for projected range change, we used the best available reported uncertainty measures (e.g., AUC SD). We acknowledge that these values reflect model‐performance uncertainty rather than direct uncertainty in projected range change and should therefore be interpreted cautiously. Furthermore, the included studies may have their own limitations related to methodological rigour, transparency, and reproducibility, which can affect the reliability and comparability of findings. In this context, future systematic reviews may benefit from evaluating transparency and reproducibility as part of their inclusion criteria, particularly by considering the availability of open‐access data, metadata, and open‐source code (Huettmann and Arhonditsis 2023).

## 4 Conclusion

Arboreal mammal species are diverse, with each species typically adapted to local climatic conditions. These adaptations create complex challenges under climate change, further influenced by varied natural and human‐induced environmental conditions across their ranges. This systematic review highlights that most research focuses on empirical and experimental approaches, while predictive modelling remains limited. In addition, current research on arboreal mammals is regionally and taxonomically biased, limiting our understanding of climate change impacts. Climate change generally has negative effects on arboreal mammals, mainly associated with extreme temperatures, with few studies noting positive effects. Most studies examined only one climate factor and a single species, neglecting the interactions among multiple climate drivers and species within shared ecosystems. Addressing these gaps requires targeted, long‐term research that integrates diverse taxa, regions, and high‐resolution data. Furthermore, incorporating a broader range of environmental predictors and adopting a multispecies approach will be essential to enhance understanding of the complex effects of climate change on arboreal mammals. However, we understand that these studies require consistent funding, proper data management, and collaboration efforts, all of which undoubtedly need strong support from decision‐makers. Given the emerging nature of climate change research, it is essential to conduct more studies integrating the above‐discussed facts. This will help to capture the varied effects of climate change on arboreal mammals and support the formulation of future conservation strategies.

## Author Contributions

**Dinayadura Kalathma Navanjalie Samanthi De Silva:** writing – original draft, visualization, formal analysis. **Carly Campbell:** writing – review and editing, supervision. **Grant Hamilton:** writing – review and editing, supervision.

## Conflicts of Interest

The authors declare no conflicts of interest.

## Supporting information


**Supplementary Material 1**.

**Supplementary Material 2**.

**Supplementary Material 3**.

## Data Availability

The data that support the findings of this study are openly available in https://doi.org/10.5281/zenodo.21413772.
