# Supplementary material for: Arboreal Mammals Are at Risk Under Climate Change: Insights From a Global Systematic Review and Meta‐Analysis
Source: Glob Chang Biol. 2026 Aug 8;32(8):e71034. doi: 10.1111/gcb.71034 (PMC13451958; doi:10.1111/gcb.71034)
Supplement: Supplementary file 1 [file GCB-32-e71034-s003.docx]

**Supplementary material 1**

# **Title:** Arboreal mammals are at risk under climate change: Insights from a global systematic review and meta-analysis

# Inclusion /Exclusion criteria

- **Inclusion Criteria:**

Focusing on at least one arboreal mammal

Peer-reviewed articles and conference papers and reviews

includes grey literature: government reports.

Not a book chapter excerpt, conference abstract

Studies that investigate direct or indirect impact of climate change on arboreal mammals

Seasonal studies may be included only if some climatic variables were measured

(No time or geographical restrictions)

- **Exclusion Criteria:**

Non-peer-reviewed articles, preprints, abstracts, book chapters.

Articles not available in English

Studies not focusing on at least one arboreal mammal

studies not related to direct or indirect impact of climate change

**Fig. 1.** PRISMA diagram showing the systematic search for literature on the impacts of climate change on arboreal mammals.

Arboreal mammal?

Excluded

No

Yes/probably

Climate change impact?

No

Excluded

Yes/probably

Included

**Fig. 2.** Decision tree methodology for screening studies in screening titles and abstracts.

# Review protocol

This systematic review was conducted following the PRISMA 2020 guidelines (Preferred Reporting Items for Systematic Reviews and Meta-Analyses) (Supplementary material 1: Fig. 1) (Page et al., 2021). We conducted a systematic search on October 24, 2024, using the Scopus and Web of Science (WoS) online databases, and repeated the search on July 10, 2025, to capture any new studies. The searches were refined by document type and language. Only English-language articles from peer-reviewed journals and proceedings were included, while book chapters, theses, and reports were excluded. There were no restrictions on the publication year.  To improve the probability of identifying the relevant papers a comprehensive search string was developed using diverse key terms related to climate change, temperature shifts, and extreme weather events addressing both direct and indirect climate impacts on arboreal mammals. Seasonal studies also were included only if some climatic variables were measured (Supplementary material 1: Inclusion exclusion criteria). The exact search strings were as follows:

Scopus:

TITLE-ABS-KEY((( (mammal* OR species OR animal) AND ( arboreal ) ) AND ( ( " climat* change" OR "global warming" OR "climat* warming" OR "environment* change" OR "changing climate" OR "Climate variability") OR ( ( increase* OR decrease* OR shift*) near/5 AND temperature* ) OR ( weather AND ( extreme OR severe ) ) OR ( wildfire* OR cyclone* OR bushfire* OR heatwave* OR drought* ) )) AND NOT ( terrestrial OR "ground dwelling" OR bird* OR reptil* OR amphibia* OR invertebrat*)) AND ( LIMIT-TO ( LANGUAGE,"English" ) ) AND ( LIMIT-TO ( DOCTYPE,"cp" ) OR LIMIT-TO ( DOCTYPE,"ar" ) OR LIMIT-TO ( DOCTYPE,"re" ) )

Web of Science:

TS=((( mammal* OR species OR animal ) AND ( arboreal ) ) AND (("climat* change" OR "global warming" OR "climat* warming" OR "environment* change" OR "changing climate" OR "climate variability") OR ((increase* OR decrease* OR shift*) NEAR/5 temperature*) OR (weather AND (extreme OR severe)) OR (wildfire* OR cyclone* OR bushfire* OR heatwave* OR drought*))) NOT TS=((terrestrial OR "ground dwelling" OR bird* OR reptil* OR amphibia* OR invertebrat*)) and Article or Review Article or Proceeding Paper (Document Types) and English (Languages)

We identified 381 studies in Scopus and 366 in Web of Science that matched the search terms, resulting in a total of 747 records. We used Covidence software to remove duplicates and to screen titles and abstracts (Covidence, 2024). After removing duplicates, we were left with 520 unique records. Following title and abstract screening, about 81% of the papers were excluded because the articles were not related to arboreal mammals or did not include any climate change impacts. The decision tree used in this context is provided in Supplementary material 1. The full texts of the remaining 90 papers were screened and excluded based on the following criteria: (1) the species was not truly an arboreal mammal (2) wrong publication type (e.g., reviews) and (3) lack of explicit connection between events and climate change as a driving factor. For instance, if the article is about wildfires (event) but does not mention changing climate as a driver, we excluded those articles from our review. Likewise, we had to exclude many articles mainly relevant to wildfires.

After reviewing the full texts, 59 papers were retained from the database searches. We further conducted backward and forward citation searching of each retained paper, as well as excluded review articles in the above step, and identified additional studies that met our inclusion criteria, resulting in a final total of 142 studies.

**Fig. 3.** Sankey diagram showing the relationship between country, family of the species and climate driver of included empirical studies

**Fig. 4**. Sankey diagram showing the relationship between country, family of the species, climate driver, and impact of included empirical studies

**Fig. 5.** Sankey diagram showing the relationship between country, family of the species, climate driver, and impact of both predicted and empirical studies

**Bibliometric Analysis**

1. Annual trends in the publication of indexed articles

**Fig. 6**. Annual scientific publication

2. Most relevant authors

**Table 1**. Most relevant authors based on the number of articles and fractionalized contributions

| **Author** | **Articles** | **Fractionalized** |
| --- | --- | --- |
| Lunney D | 9 | 2.34 |
| Rhodes JR | 7 | 1.34 |
| Lindenmayer DB | 6 | 1.1 |

3. Most relevant sources

**Table 2.** Most relevant journals identified in the bibliometric analysis

| **Source** | **Articles** |
| --- | --- |
| PLOS ONE | 7 |
| Biological Conservation | 6 |
| Journal of Mammalogy | 6 |
| Wildlife Research | 6 |
| Global Change Biology | 5 |
| Scientific Reports | 5 |
| American Journal of Primatology | 4 |
| Australian Zoologist | 4 |
| Diversity and Distributions | 4 |
| Ecography | 4 |
|  |  |

4. The contribution of corresponding authors by country

**Table 3**. Country contributions based on the corresponding author's affiliation

| **Country** | **Articles** | **Freq** | **SCP** | **MCP** | **MCP_Ratio** |
| --- | --- | --- | --- | --- | --- |
| Australia | 58 | 0.4677 | 52 | 6 | 0.103 |
| Brazil | 14 | 0.1129 | 9 | 5 | 0.357 |
| USA | 9 | 0.0726 | 7 | 2 | 0.222 |
| Finland | 6 | 0.0484 | 4 | 2 | 0.333 |
| China | 5 | 0.0403 | 3 | 2 | 0.4 |
| Mexico | 5 | 0.0403 | 3 | 2 | 0.4 |
| South Africa | 5 | 0.0403 | 1 | 4 | 0.8 |
| Canada | 2 | 0.0161 | 1 | 1 | 0.5 |
| Chile | 2 | 0.0161 | 2 | 0 | 0 |
| India | 2 | 0.0161 | 2 | 0 | 0 |

**Fig. 7**. Most productive countries

5. International collaboration networks

**Fig. 8**. Country- level international collaboration network identified in the bibliometric analysis.

6. The most cited global articles.

**Table 4.** Top globally cited articles based on total citations, citations per year, and normalised citation impact

| **Paper** | **DOI** | **Total Citations** | **TC per Year** | **Normalized TC** |
| --- | --- | --- | --- | --- |
| KEARNEY MR, 2010, CONSERV LETT | 10.1111/j.1755-263X.2010.00097.x | 406 | 25.38 | 3.21 |
| WELBERGEN JA, 2008, PROC R SOC B BIOL SCI | 10.1098/rspb.2007.1385 | 358 | 19.89 | 3.30 |
| SEDGELEY JA, 2001, J APPL ECOL | 10.1046/j.1365-2664.2001.00607.x | 221 | 8.84 | 1.00 |
| LINDENMAYER DB, 2011, BIOL CONSERV | 10.1016/j.biocon.2011.02.022 | 137 | 9.13 | 1.54 |
| GARROWAY CJ, 2010, GLOBAL CHANGE BIOL | 10.1111/j.1365-2486.2009.01948.x | 132 | 8.25 | 1.04 |
| ADAMS RA, 2010, ECOLOGY | 10.1890/09-0091.1 | 121 | 7.56 | 0.96 |
| BRISCOE NJ, 2016, GLOB CHANG BIOL | 10.1111/gcb.13280 | 102 | 10.20 | 2.97 |
| BRISCOE NJ, 2014, BIOL LETT | 10.1098/rsbl.2014.0235 | 96 | 8.00 | 2.05 |
| GORDON G, 1988, AUST J ECOL | 10.1111/j.1442-9993.1988.tb00993.x | 96 | 2.53 | 1.00 |
| SEABROOK L, 2011, WILDL RES | 10.1071/WR11064 | 95 | 6.33 | 1.07 |
|  |  |  |  |  |

7. Co-citation network analysis

**Fig. 9**. Co-citation network identified from the bibliometric analysis
